# Pooled Sequencing Analysis of Geese (*Anser cygnoides*) Reveals Genomic Variations Associated With Feather Color

**DOI:** 10.3389/fgene.2021.650013

**Published:** 2021-06-18

**Authors:** Shuang Ren, Guangqi Lyu, David M. Irwin, Xin Liu, Chunyu Feng, Runhong Luo, Junpeng Zhang, Yongfeng Sun, Songyang Shang, Shuyi Zhang, Zhe Wang

**Affiliations:** ^1^College of Animal Science and Veterinary Medicine, Shenyang Agricultural University, Shenyang, China; ^2^College of Food Science, Shenyang Agricultural University, Shenyang, China; ^3^Department of Laboratory Medicine and Pathobiology, University of Toronto, Toronto, ON, Canada; ^4^College of Animal Science and Technology, Jilin Agricultural University, Changchun, China

**Keywords:** goose, feather color, genome, pool-seq, SNP

## Abstract

During the domestication of the goose a change in its feather color took place, however, the molecular mechanisms responsible for this change are not completely understood. Here, we performed whole-genome resequencing on three pooled samples of geese (feral and domestic geese), with two distinct feather colors, to identify genes that might regulate feather color. We identified around 8 million SNPs within each of the three pools and validated allele frequencies for a subset of these SNPs using PCR and Sanger sequencing. Several genomic regions with signatures of differential selection were found when we compared the gray and white feather color populations using the *F*_ST_ and *Hp* approaches. When we combined previous functional studies with our genomic analyses we identified 26 genes (*KITLG, MITF, TYRO3, KIT, AP3B1, SMARCA2, ROR2, CSNK1G3, CCDC112, VAMP7, SLC16A2, LOC106047519, RLIM, KIAA2022, ST8SIA4, LOC106044163, TRPM6, TICAM2, LOC106038556, LOC106038575, LOC106038574, LOC106038594, LOC106038573, LOC106038604, LOC106047489, and LOC106047492*) that potentially regulate feather color in geese. These results substantially expand the catalog of potential feather color regulators in geese and provide a basis for further studies on domestication and avian feather coloration.

## Introduction

**FIGURE 1:**
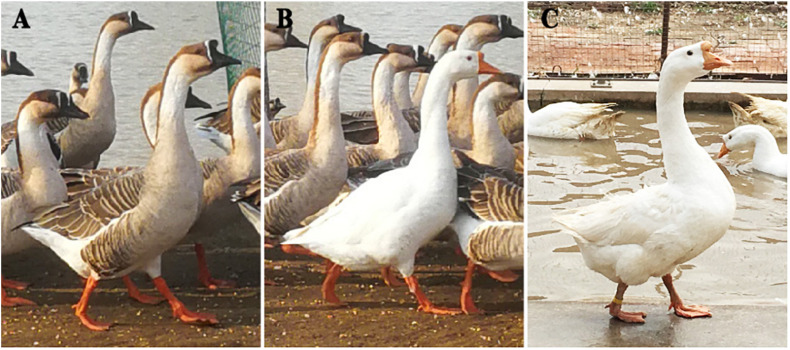
Images of swan geese (*Anser cygnoides*) sequenced in this study showing their feather color phenotypes. **(A)** Feral gray geese (group gray) have the same feather phenotype as wild geese. **(B)** A feral white goose (group White_1) living in a population of feral gray geese. **(C)** Domesticated white geese (group White_2).

Through more than 5000 years of constant artificial selection, domesticated geese have acquired a number of modifications to their appearance compared to their wild ancestors and relatives (Zeuner, 1963; Albarella, 2005). Most Asian and some European domestic goose breeds were derived from the swan goose (*Anser cygnoides*) (Buckland and Gérard, 2002). Domestication involved a complex set of metabolic, physiological and behavioral changes, including traits involving the liver, meat, eggs and feathers, but the most visible difference between wild and domestic swan geese is their feather coloration (Sossinka, 1982). Wild swan geese are characterized by their iconic feathers with gray stripes (Figure 1A), while domestic swan geese have an all-white color appearance (Figures 1B,C; Gao et al., 2016).

Since feather coloration and patterns are prominent features in birds, and play essential roles in their survival, mechanisms that regulate the differentiation of feather color has been intensively studied (Abolins-Abols et al., 2018; Gao et al., 2018). Feather color is the consequence of two different, but related, physical processes, pigmentation and structural coloration, where pigmentation is the primary basis for the color diversity in animals (D’Alba et al., 2012). Melanins and carotenoids are widely distributed pigments in avian feathers and are the main contributors to the diversity of feather color in birds. The melanin content is usually higher than that of carotenoids, where studies have shown that the melanin content of feathers in swallows is four orders of magnitude greater than that of carotenoids (McGraw et al., 2004; McGraw, 2006; Delhey, 2015). The genetic control of melanogenesis in birds is achieved through genes that encode specific enzymes involved in melanin synthesis as well as other regulatory and structural proteins required for the distribution of melanin (Galván and Solano, 2016).

Investigations in some avian species have identified a limited number of genes involved in the mechanisms controlling feather coloration, however, only a few studies have focused on changes in feather color in geese or swans (Theron et al., 2001; Mundy et al., 2004; Bed’hom et al., 2012; Emaresi et al., 2013; Wang et al., 2014; Poelstra et al., 2015; Zhou et al., 2018; Wen et al., 2021). Melanic plumage polymorphisms in the lesser snow geese (*Anser caerulescens caerulescens*) and arctic skuas (*Stercorarius parasiticus*) correlate with changes in the copy number of variant *MC1R* alleles (Mundy et al., 2004). In the black and black-necked swans (*Cygnus atratus* and *C. melanocoryphus*), independently derived nucleotide substitutions in *MC1R*, which cause amino acid changes at important functional sites, have been identified that are consistent with increased MC1R activity and melanism pigment synthesis (Pointer and Mundy, 2008). In the domestic swan geese, three SNPs in *TYR* and one in *MITF* have been reported to be associated with white plumage (Wang et al., 2014). Recently, Wen et al. (2021) reported, in a genomic level examination of plumage color in domestic geese, an 18 bp deletion in an intron region of *KIT* (NW_013185664.1, 11,785,718–11,785,736 bp) that was associated with white feather color.

Although *TYR*, *MITF* and *KIT* have been found to be associated with differences in feather coloration in domestic geese (Wang et al., 2014; Wen et al., 2021), a full understanding of the genetic basis of feather color formation in this species remains incomplete. With the unprecedented development of high-throughput sequencing, it has become possible to examine the genetic basis of differences in feather color at the genomic level (Zhou et al., 2018).

In this study, we performed whole-genome pooled sequencing (Pool-Seq) on three populations of swan geese with wild type and white-colored feathers. By identifying genomic regions that experienced selective sweeps, we aimed to identify genes that have experienced artificial selection and thus might explain the change in feather color in domesticated geese.

## Materials and Methods

### Whole-Genome Pooled Sequencing of Goose DNA Samples

A total of 117 feral gray (*Anser cygnoides*, 60 females, 57 males, group gray; Figure 1A), 25 feral white (*Anser cygnoides*, 10 females, 15 males, group White_1; Figure 1B), and 87 domesticated white (*Anser cygnoides domesticus*, 52 females, 35 males, group White_2; Figure 1C) geese were sampled. These samples were collected from large populations to minimize genetic relationships. We choose our sample sizes for this Pool-seq study based on previous reports on Darwin’s finch (sample size range 8–35) and monarch butterflies (sample size range 9–101) (Zhan et al., 2014; Lamichhaney et al., 2015). The accuracy of Pool-Seq increases with larger numbers of individuals included in the pool (Futschik and Schlötterer, 2010; Gautier et al., 2013). This suggests that our samples should be sufficient to identify SNPs and genes associated with feather color. A subset of SNPs identified from Pool-Seq were validated by Sanger sequencing to assess the accuracy of estimating allele frequencies (AFs) using Pool-Seq. Blood samples were collected by venipuncture. Gray and White_1 geese were acquired from a population maintained at the Xianghai breeding base in Jilin city, Jilin province, China. White_2 geese, belonging to the Huoyan breed, were obtained from the Liaoyang Animal Science Research Institute, Liaoning province, China. Geese in the group gray, with wild-type feather color, were the offspring of a mating between a population of male wild geese and female domestic geese. After several generations of breeding, a sub-population of feral white (White_1) geese appeared among the feral gray geese. Although fed by humans, unlike the domesticated white geese (White_2), both the feral gray and feral white geese possess flight abilities similar to those of wild geese, which is considered to be a signature of feralization (Gering et al., 2019).

Genomic DNA was extracted individually from blood samples of each goose using a Blood Genome DNA Extraction Kit (TIANGEN, DP348) following the manufacturer’s instructions. Equimolar quantities (3 μg/ml) of DNA from each individual were pooled to establish the three sequencing libraries. The first pooled sample was from 117 feral gray geese, the second from 25 feral white geese and the third from 87 domestic white geese. The concentrations and purity of genomic DNA were checked before library construction. Libraries were generated via adapter ligation and DNA cluster preparation and subjected to 150 bp paired-end sequencing on an Illumina HiSeq 4000 platform. Sequencing depth of each library was at least 30×. Library construction and genome sequencing was conducted by the Beijing Genomics Institute Co., Ltd. (Shenzhen, China).

### Data Processing, Mapping and SNP Calling

We applied the PoolParty pipeline (Micheletti and Narum, 2018), which was designed for pool sequencing, to analyze the sequence data. The module PPAlign was used to align each read to the reference genome and for SNP calling. The parameters of module PPAlign were: “THREADZ = 32 BQUAL = 20 MAPQ = 5 SNPQ = 20 MINLENGTH = 25 INWIN = 3 MAF = 0.05 KMEM = Xmx4g MINDP = 10”. Briefly, BBDuk^1^ was used to obtain clean data by trimming primer dimers and adapter sequences from the reads, discarding bases with quality lower than Q20 and reads with lengths less than 25 bp. BWA-MEM (Li and Durbin, 2009) was then used to map the clean data to the goose reference genome (AnsCyg_PRJNA183603_v1.0)^2^ (Lu et al., 2015).

Prior to SNP calling, SAMBLASTER^3^ was used to mark duplicate read pairs and compress the alignment to eliminate any bias generated during the PCR amplification for library preparation and/or sequencing (Faust and Hall, 2014). Aligned results were then sorted by Picard Tools^4^ () and ambiguously mapped or unaligned reads were removed with SAMtools (Li et al., 2009). BCFtools (Li, 2011) was then used to call and filter the SNPs into a VCF file. Filtered alignments were combined in mpileup format for downstream analyses. SNPs with sequencing depth < 10 folds, quality < 20, minor allele frequency (MAF) < 0.05 or within 15 bp of indel were discarded.

### Variant Discovery and SNP Annotation

SNP annotation and the functional consequences of sequence variants were predicted using the Ensembl Variant Effect Predictor (VEP) tool using Ensembl database version 103 with the input VCF file (McLaren et al., 2016). Annotated results of VEP included transcripts, proteins, regulatory regions, and phenotype (McLaren et al., 2016). We grouped loss-of-function (LoF) variants into four categories (1, stop-gain and stop-loss; 2, frameshift indel; 3, donor and acceptor splice-site; and 4, initiator codon variants) (Sveinbjornsson et al., 2016). Marker coverage for each gene included 10 kb of upstream and downstream flanking region (Potter et al., 2010). We focused on LoF variant annotation results for the downstream analysis.

### Sanger Sequencing Validation of SNP Allele Frequencies (AFs)

SNP AFs were calculated from the read depths of each allele in the Pool-Seq data. To confirm the accuracy of AFs estimated from the Pool-Seq data, we performed a Kendall W’s coordination coefficient test on a subset of the SNPs (28 loci) (Dodge and Commenges, 2006). Of these SNPs, 15 SNPs (SNP01, SNP06 and SNP11-23 in Supplementary Table 1) were selected as they had the lowest *P*-values in the comparison of the Gray and White_2 groups by Fisher’s exact test based on read depth of alleles. Eight SNPs (SNP02-05 and SNP7-10 in Supplementary Table 1) were selected as they were adjacent to SNP01 and SNP06 and could be amplified with the same primer pairs used for them. Five SNPs (SNP24-28 in Supplementary Table 1) located in four genes (*KITLG*, *MITF*, *TYRO3*, and *KIT*) were also selected as these genes had previously been reported to be associated with the regulation of feather or coat color (Wehrle-Haller, 2003; Zhu et al., 2009; Zhou et al., 2018; Wu et al., 2019). The SNP alleles selected for validation were genotyped in all 229 individual geese by Sanger sequencing and the AFs were calculated from the genotype data. The two estimates of AFs, which were obtained from Pool-Seq and Sanger sequencing data, were compared using the Kendall W’s coordination coefficient test. Chi-square tests were performed to test the significance of the associations between the five SNPs in the color-related genes (*KITLG*, *MITF*, *TYRO3*, and *KIT*) and feather color phenotype. Primers used for the amplification of the selected SNPs are listed in Supplementary Table 1.

### Detection of Selective Sweeps

To accurately detect genomic regions in geese that had experienced selection during domestication and to estimate the patterns of genetic diversity across the goose genome, we conducted selective sweep analyses including the fixed index (*F*_ST_) and pooled heterozygosity (*Hp*) approaches (Rubin et al., 2010; Micheletti and Narum, 2018). *F*_ST_ in 10-kb non-overlapping sliding windows were calculated using the “fst-sliding.pl” module in Popoolation2 (Kofler et al., 2011), according to Weir and Cockerham’s method (Weir and Cockerham, 1984). The global parameters of *F*_ST_ approach were: “MINCOV = 10 MAXCOV = 100 MAF = 0.05.” *Hp* and negative Z-transformed *Hp* (−ZHp) were calculated using a custom python3 script in 10-kb non-overlapping sliding windows. The *Hp* approach determines, for each pool and SNP, the numbers of reads corresponding to the most (*n*_*MAJ*_) and least (*n*_*MIN*_) abundant alleles. For each window in each breed pool, the heterozygosity score of the pool was calculated as:

$$H\,p=\frac{2\,\sum n_{M\,A\,J}\,\sum n_{M\,I\,N}}{{\left(\sum n_{M\,A\,J}+\sum n_{M\,I\,N}\right)}^{2}}$$

Where *n*_*MAJ*_ and *n*_*MIN*_ represent the numbers of reads corresponding to the most and least abundant allele. Individual *Hp* values were then Z-transformed as follows:

$$-\mathrm{ZHp}=-\frac{H\,p-\mu \,H\,p}{\sigma \,H\,p}$$

Windows with less than 10 SNPs were discarded to avoid spurious signals. Windows located in the top 3% of the *F*_ST_ distribution and top 3% of the −ZHp distribution were regarded as candidate regions for selective sweeps (Wang et al., 2015). Genes overlapping these regions were identified using Ensembl genome annotation.

Genomic regions that might have experienced selective sweeps were identified through three steps: (1) windows in the top 3% of the *F*_ST_ distributions of both the Gray vs. White_1 and the Gray vs. White_2 comparisons were identified; (2) windows in the top 3% of the −ZHp distributions of both the White_1 and the White_2 populations were identified; (3) the intersection of region identified in (1) and (2) were considered to have experienced a selective sweep. Genes located in these overlapped regions might be involved in the change of goose feather color.

### Gene Ontology (GO) and KEGG Pathway Enrichment Analysis

To determine the possible function of genes that were located in the selective sweep regions, we identified orthologous human genes using the BioMart online tool^5^. The orthologous genes were then uploaded into the DAVID online tool to test for enrichment in gene ontology (GO) terms (Huang et al., 2009). KEGG pathway analysis was conducted using the online KOBAS tool (Xie et al., 2011). A Fisher’s exact test was then used to determine the significance of the enrichments of the GO terms and KEGG pathways, with a significant level of *P* < 0.05.

## Results

### Statistics of the Genome Resequencing Data

A total of 148.26 Gb clean data was obtained from the three Pool-Seq libraries (Table 1). Mapping rates for the libraries varied between 98.14 and 98.23%, with the final effective mapping depths ranging from 44.09- to 44.13-fold. The Q20 rates for the three libraries were all over 98%. An average of 8,476,172 SNPs was identified in each library.

**TABLE 1:** Summary statistics of the clean data from whole-genome resequencing.

Parameter	Gray	White_1	White_2
Clean data (Gb)	49.42	49.40	49.44
Reads (M)	329.45	329.30	329.59
Map reads rate (%)	98.14	98.20	98.23
Q20 rate (%)	98.08	98.14	98.17
Sequencing depth	44.11	44.09	44.13
Total SNPs	8,785,296	8,680,731	7,962,489

### Sanger Sequencing Validation

To assess the reliability of estimating allele frequencies of SNPs using the population genomic sequencing (Pool-Seq) data, we genotyped 28 SNPs from an average of 210 individuals using Sanger sequencing (Supplementary Tables 2, 3). AFs calculated from the Sanger sequencing data, based on individual amplifications and sequencing, were in accord with the AFs calculated from the Pool-Seq data. Kendall W’s coordination coefficients for the comparisons of the AFs estimated from the Pool-Seq and Sanger genotypes for the Gray, White_1 and White_2 populations were 0.96, 0.97 and 0.94 (*P* < 0.05), respectively, showing that there is a good concordance between the results obtained using the two different methods.

Among the 28 SNPs examined above, five were SNPs that are located in four genes (*KITLG*, *MITF*, *TYRO3*, and *KIT*) previously reported to be associated with feather or coat color (Wehrle-Haller, 2003; Zhu et al., 2009; Zhou et al., 2018; Wu et al., 2019). Of these five SNPs, two are located in the 3′ UTR of *KITLG* (NW_013185706.1: G232853A and NW_013185706.1: C232854T), one in the 5′ UTR of *MITF* (NW_013185692.1: G4400553C), one in the *TYRO3* (S772G) coding region and one in the *KIT* (T887A) coding region (Table 2). Results from a Chi-square test showed an extremely significant association between the SNP genotypes and feather color phenotypes (*P* < 0.001) for these SNPs.

**TABLE 2:** Genotypes of the five PCR verified loci.

SNP information^a^	Gene	Genotype	Numbers of individuals^b^			χ^2^ value
			
			Gray	White_1	White_2	
NW_013185706.1: 3′UTR_G232853A	*KITLG*	*GG*	37	22 (88%)	84 (100%)	102.89
		*GA*	77 (68%)	3	0	(*P* < 0.001)
		*AA*	0	0	0	
NW_013185706.1: 3′UTR_C232854T	*KITLG*	*CC*	37	22 (88%)	84 (100%)	102.89
		*CT*	77 (68%)	3	0	(*P* < 0.001)
		*TT*	0	0	0	
NW_013185692.1: 5′UTR_G4400553C	*MITF*	*GG*	83 (74%)	6	14	80.44
		*GC*	29	17 (68%)	49 (59%)	(*P* < 0.001)
		*CC*	0	2	20	
NW_013185657.1: cds_A2638G:S772G	*TYRO3*	*AA*	31	20 (80%)	86 (100%)	106.57
		*AG*	76 (71%)	5	0	(*P* < 0.001)
		*GG*	0	0	0	
NW_013185664.1: cds_A2659G:T887A	*KIT*	*AA*	0	0	0	93.80
		*AG*	67 (66%)	2	0	(*P* < 0.001)
		*GG*	35	23 (92%)	78 (100%)	

*^*a*^SNP information is displayed in the following format: NCBI GenBank accession number: nucleotide position in the goose reference genome (cds, 5*′*UTR or 3*′*UTR) + nucleotide in the reference genome (reference nucleotide) + SNP position in the transcript + mutant nucleotide: amino acid in the goose reference transcript + codon position in the transcript + amino acid mutation or no change.*

*^*b*^Numbers of individuals (Gray| White_1| White_2) for which reliable genotypes could be inferred from the Sanger sequencing data. The percentage in parenthesis is the percentage of the genotype in the number of verified individuals.*

### Selective Sweep Analysis

**FIGURE 2:**
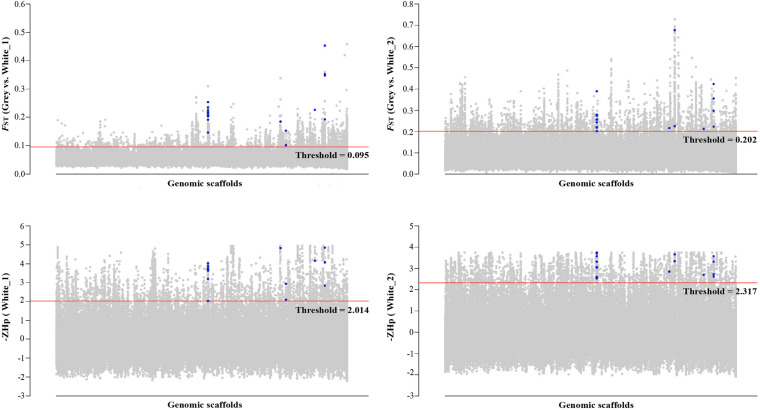
Genomic landscape of the signatures of positive selection detected using the *F*_ST_ and −ZHp approaches. *F*_ST_ and −ZHp thresholds (cutoff 3%) are represented as red lines. Blue dots represent the 17 candidate genes in the overlapping windows from the *F*_ST_ and *Hp* methods. Genomic scaffolds represent the complete goose genome composed of multiple scaffolds.

*Hp* and −ZHp distributions are presented in Supplementary Figure 1. The selective sweep analyses identified (1) 317 regions in the top 3% of the *F*_ST_ distributions of the intersection of the Gray vs. White_1 (*F*_ST_ value, mean = 0.119, range 0.095–0.457) and the Gray vs. White_2 (*F*_ST_ value, mean = 0.258, range 0.202–0.727) comparisons and (2) 253 regions in the top 3% of the −ZHp distributions of the intersection of the White_1 (−ZHp value, mean = 2.835, range 2.014–4.952) and White_2 (−ZHp value, mean = 2.904, range 2.317–3.755) populations (Figure 2). A total of 99 genes were identified in the 317 regions identified by the *F*_ST_ distributions and 103 genes identified in the 253 regions identified by the −ZHp distributions (Supplementary Tables 4, 5). Among the 99 genes identified from the *F*_ST_ distributions, four (*SLC16A2*, *AP3B1, SMARCA2*, and *VAMP7*) have previously been associated with animal coloration (Table 3). Similarly, 5 of the 103 genes from the −ZHp distributions (*SLC16A2*, *ROR2, CSNK1G3, CCDC112*, and *VAMP7*) were previously associated with animal coloration (Table 3).

**TABLE 3:** Genes associated with animal coloration that overlap with the selected regions under detected by the *F*_ST_ or *Hp* approach.

Method	Scaffold^a^	Gene symbol	Summary of gene function
*F*_ST_ (Top 3%)	NW_013185770.1	*AP3B1*	Melanin formation (Jing et al., 2014)
	NW_013185722.1	*SLC16A2*	Pigment-related (Baxter et al., 2019)
	NW_013185909.1	*SMARCA2*	Melanin formation (Mehrotra et al., 2014)
	NW_013185915.1	***VAMP7***	Melanin formation (Yatsu et al., 2013)
*Hp* (Top 3%)	NW_013185722.1	*SLC16A2*	Pigment-related (Baxter et al., 2019)
	NW_013185840.1	*ROR2*	Melanin formation (O’Connell et al., 2013)
	NW_013185881.1	*CSNK1G3*	Pigment-related (Al Robaee et al., 2020)
	NW_013185883.1	***CCDC112***	Pigment-related (Tian et al., 2014)
	NW_013185915.1	***VAMP7***	Melanin formation (Yatsu et al., 2013)

*^*a*^AnsCyg_PRJNA183603_v1.0 primary assembly.*

*Gene names in bold have LoF variation according to VEP annotation.*

More importantly, 27 regions, which included 17 genes, were found in the overlap of the 317 *F*_ST_ and 253 −ZHp top 3% regions, including two, *SLC16A2* and *VAMP7* that had previously been associated with animal coloration (Table 4). The 15 novel genes identified here are: *LOC106038556*, *LOC106038575*, *LOC106038574*, *LOC106038594*, *LOC106038573*, *RLIM*, *LOC106038604*, *KIAA2022*, *ST8SIA4*, *LOC106044163*, *TRPM6*, *TICAM2*, *LOC106047489*, *LOC106047492*, and *LOC106047519* (Table 4). LoF (loss of function) variants were found for 9 of the genes (8 detected by both the *F*_ST_ and the *Hp* approaches (*LOC106038574*, *LOC106038604 LOC106044163*, *TICAM2*, *LOC106047489*, *VAMP7*, *LOC106047492*, and *LOC106047519*) and one by only *Hp* (*CCDC112*) in the selective sweep regions of geese with different feather colors (or in the 10 kb region upstream and downstream of the sweep regions) (Supplementary Table 6).

**TABLE 4:** 17 genes found in the overlapping regions identified by both the *F*_ST_ and the *Hp* differentiation approaches.

Scaffold^a^	Position^b^	Gene symbol	Gene description
NW_013185722.1	261,870	*LOC106038556*	Homeobox protein CDX-4-like
NW_013185722.1	288,464	*LOC106038575*	Uncharacterized LOC106038575
NW_013185722.1	301,490	***LOC106038574***	Pre-mRNA 3′-end-processing factor FIP1-like
NW_013185722.1	338,779	*LOC106038594*	Ligand of Numb protein X 2-like
NW_013185722.1	371,156	*LOC106038573*	Uncharacterized LOC106038573
NW_013185722.1	502,677	*SLC16A2*	Solute carrier family 16 member 2
NW_013185722.1	517,620	*RLIM*	Ring finger protein, LIM domain interacting
NW_013185722.1	537,179	***LOC106038604***	Uncharacterized LOC106038604
NW_013185722.1	548,033	*KIAA2022*	Neurite extension and migration factor
NW_013185807.1	123,1519	*ST8SIA4*	ST8 alpha-N-acetyl-neuraminide alpha-2,8-sialyltransferase 4
NW_013185817.1	975,069	***LOC106044163***	Proprotein convertase subtilisin/kexin type 5-like
NW_013185817.1	143,0086	*TRPM6*	Transient receptor potential cation channel subfamily M member 6
NW_013185883.1	304,453	***TICAM2***	Toll like receptor adaptor molecule 2
NW_013185915.1	689,355	***LOC106047489***	SLAIN motif-containing protein-like
NW_013185915.1	717,937	***VAMP7***	Vesicle associated membrane protein 7
NW_013185915.1	737,829	***LOC106047492***	DNA-directed RNA polymerases I and III subunit RPAC2-like
NW_013185915.1	745,589	***LOC106047519***	Endothelin B receptor-like

*^*a*^Same as Table 3.*

*^*b*^Start position of the gene in the genome.*

*Eight gene names in bold have LoF variants according to VEP annotation.*

### GO and KEGG Pathway Enrichment Analysis

We conducted GO and KEGG pathway analyses of the 17 genes identified in the selective sweep regions. These genes were found to be significantly enriched for the GO term “late endosomal membrane category” (GO: 0031902, *P* < 0.05) and in three pathways, “RNA polymerase,” “SNARE interactions in vesicular transport” and “cytosolic DNA-sensing pathway” (*P* < 0.05, Supplementary Table 7).

## Discussion

In this study, we performed whole-genome Pool-Seq on three populations of geese with two different colors of feathers to identify SNPs, and genes that might be responsible for these differing phenotypes. The Kendall W’s coefficients for the AFs calculated from the Pool-Seq and Sanger sequencing data indicated a good correlation between them, which suggests that our Pool-Seq data is adequate for identifying loci that are differentiated between the goose phenotypes. Selective sweep analyses of this SNP data was used to identify genomic regions that show signatures of selection during the domestication of geese. This lead to the identification of 17 genes located in candidate regions identified by both the *F*_ST_ and *Hp* approaches, suggesting a high probability that selection occurred on these genes and that they might be associated with the change in feather color seen in these geese. VEP annotation of these 17 genes identified eight with loss-of-function (LoF) alleles potentially involved in regulating feather color.

Among the 17 identified genes (Table 4), three (*VAMP7*, *SLC16A2*, and *LOC106047519*) have previously been associated with the regulation of coat color in animals (Imokawa et al., 1992; Yatsu et al., 2013; Baxter et al., 2019). *VAMP7*, vesicle associated membrane protein 7, is localized to Tyrp1-containing vesicles/organelles and acts as part of the SNARE machinery with syntaxin-3 and SNAP23 on melanosomes to regulate Tyrp1 transport in mouse melanocytes (Yatsu et al., 2013). *VAMP7* may play a key role in melanin formation and thus influence goose feather color. *SLC16A2* has an effect on pigmentation phenotypes in the zebrafish, and has the GO term “pigmentation” annotated in the Zebrafish Information Network^6^ database (Baxter et al., 2019). *LOC106047519* belongs to the ETB-R gene family, which also includes the Endothelin B receptor (*EDNRB*), and has been described as an *EDNRB*-like gene (Kanehisa, 1997; Kanehisa and Goto, 2000). *EDNRB* is reported to be associated with the development of cells of the melanocytic lineage (Imokawa et al., 1992), suggesting that *LOC106047519* might also perform a function similar to *EDNRB* to regulate feather color. Our VEP analysis identified LoF mutations in *VAMP7* and *LOC106047519*, but not in *SLC16A2*. These results suggest that *VAMP7* and *LOC106047519* might not only regulate pigmentation in the previously investigated animals but also play a role in the change in feather color in the goose.

Using either the *F*_ST_ or the *Hp* approach we identified five other genes (*AP3B1*, *SMARCA2*, *ROR2*, *CSNK1G3*, and *CCDC112*) that have been reported to be associated with animal coloration (Table 3). Substitutions in *AP3B1* cause distinct phenotypes in the pigmented cells in mouse eyes and possibly plays a role in organelle biogenesis associated with melanosomes (Jing et al., 2014). *SMARCA2*, a member of the SWI/SNF family, is involved in melanocyte differentiation and melanoma (Mehrotra et al., 2014; Markiewicz and Idowu, 2020). *ROR2* is involved in the formation of melanoma in humans, suggesting a role in melanin formation (O’Connell et al., 2013). Expression of *CSNK1G3*, a gene related to human vitiligo, is significantly reduced in C57BL/6 black mice with tyrosinase-induced depigmented skin (Ocampo-Candiani et al., 2018; Al Robaee et al., 2020). *CCDC112* regulates pigmentation and the expression level of this gene differs between Silkie and White Leghorn chickens (Tian et al., 2014). We found a LoF mutation in *CCDC112* in gray that might partly explain the difference in feather color in geese. Our results suggest that it is possible that these five genes also affect feather color in geese.

We also focused on four genes (*KITLG*, *MITF*, *TYRO3*, and *KIT*) that were previously reported to be associate with feather color (Wehrle-Haller, 2003; Zhu et al., 2009; Zhou et al., 2018; Wu et al., 2019). The SNP genotypes for these genes were also validated by Sanger sequencing (Table 2). A changes in an untranslated region (UTRs) can lead to changes in the expression of genes (Barrett et al., 2012). Here, we identified three SNPs, two located in the 3′ UTR of *KITLG* and one in the 5′ UTR of *MITF*, which are significantly associated with feather color phenotypes in our geese. This suggests that these three SNPs affect the expression of *KITLG* and *MITF* resulting in a change in feather color. Non-synonymous mutations are more likely to affect the biological function of a gene. Here, we identified two non-synonymous substitutions in *KIT* (T887A) and *TYRO3* (S772G) that are significantly associated with feather color phenotypes, indicating that they may regulate goose feather color.

GO and KEGG enrichment analyses of the 17 candidate genes in the most significant sweep areas are significantly enriched in the GO term late endosome membrane (Supplementary Table 7). Two of the candidate genes (*VAMP7* and *TICAM2*) are associated with this term. We also identified three pathways (RNA polymerase, SNARE interactions in vesicular transport and cytosolic DNA-sensing pathway) that are significantly enriched, where *VAMP7* is also involved with SNARE interactions in vesicular transport. Although the enriched GO term and the pathways do not seem to directly correlate with animal coloration, it is still possible that the genes involved in them could regulate feather coloration in geese.

In conclusion, we identified 26 genes (17 detected by both the *F*_ST_ and *Hp* approaches, five by either *F*_ST_ or *Hp* and four previously reported color-related genes) from our genomic Pool-Seq data that might be responsible for the change in feather color that occurred during the domestication of geese (*Anser cygnoides*). Among these 26 genes, 12 have previously been found to be associated with animal coloration in other studies. The roles of the other genes in feather coloration requires further investigation. Additional studies, including functional experimentation, are needed to confirm the roles of these genes, and the consequence of the mutations caused by the SNPs, on phenotypic variation in feather color in geese.

## Data Availability Statement

Whole-genome sequencing data reported in this study were deposited into the NCBI Sequence Read Archive under the accession number PRJNA532466 (https://www.ncbi.nlm.nih.gov/bioproject/PRJNA532466/).

## Ethics Statement

The animal study was reviewed and approved by the Animal Care and Use Committee of Shenyang Agricultural University. Written informed consent was obtained from the owners for the participation of their animals in this study.

## Author Contributions

SR, XL, CF, and RL performed the experiments. SR and GL analyzed the data. JZ, YS, and SZ collected the samples. SR, GL, SS, DI, and ZW wrote the manuscript. SZ and ZW designed the study and supervised the work. All authors contributed to the article and approved the submitted version.

## Conflict of Interest

The authors declare that the research was conducted in the absence of any commercial or financial relationships that could be construed as a potential conflict of interest.

## Footnotes

1 http://sourceforge.net/projects/bbmap/

2 https://www.ncbi.nlm.nih.gov/genome/?term=Anser+cygnoides

3 https://github.com/GregoryFaust/samblaster

4 http://broadinstitute.github.io/picard/

5 http://www.ENSEMBL.org/biomart/martview/

6 https://zfin.org
