# Supplementary material for: Pooled Sequencing Analysis of Geese (Anser cygnoides) Reveals Genomic Variations Associated With Feather Color
Source: Front Genet. 2021 Jun 18;12:650013. doi: 10.3389/fgene.2021.650013 (PMC8249929; doi:10.3389/fgene.2021.650013)
Supplement: Supplementary file 8 [file Data_Sheet_1.PDF]

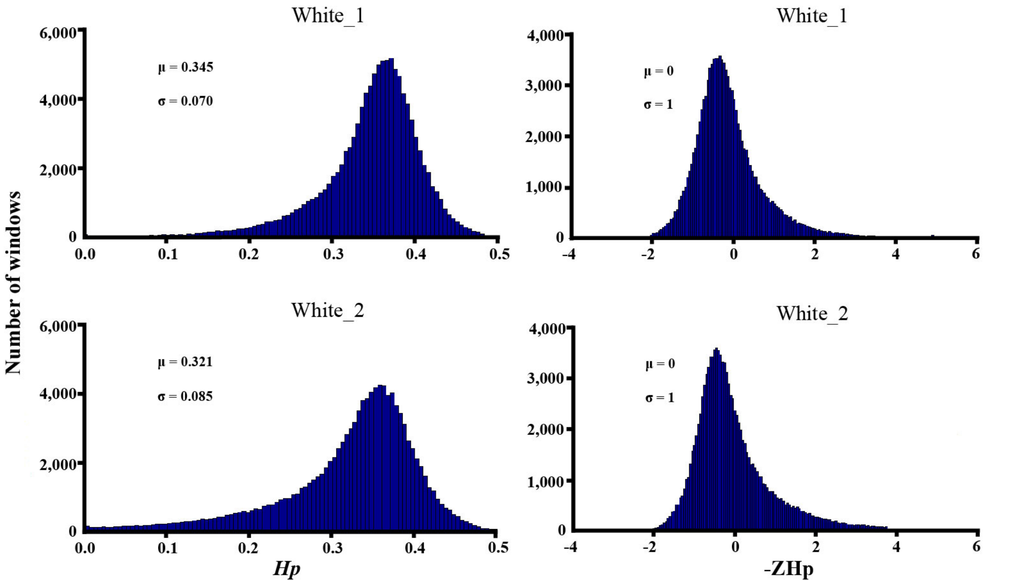

Supplementary FIGURE S1 | Histograms of genome-wide Heterozygosity values (*Hp*) and negative Z-transformed *Hp* (-ZHp) based on all 10 kb windows in groups White_1 and White_2. Bins of *Hp* and –ZHp are distributed along the x-axes. μ , mean. σ , standard deviation.
